# Nucleus Accumbens Shell Dopamine Preferentially Tracks Information Related to Outcome Value of Reward

**DOI:** 10.1523/ENEURO.0058-17.2017

**Published:** 2017-06-07

**Authors:** Deirdre A. Sackett, Michael P. Saddoris, Regina M. Carelli

**Affiliations:** 1 Department of Psychology and Neuroscience, University of North Carolina, Chapel Hill, NC 27599; 2 Department of Psychology and Neuroscience, University of Colorado Boulder, Boulder, CO 80309

**Keywords:** Accumbens, behavior, decision making, dopamine, electrochemistry, reward

## Abstract

Effective decision-making requires organisms to predict reward values and bias behavior toward the best available option. The mesolimbic dopamine system, including the nucleus accumbens (NAc) shell and core, is involved in this process. Although studies support a role of the shell and core in specific aspects of decision-making (e.g., risk, effort, delay), no studies have directly compared dopamine release dynamics in these subregions to cues exclusively signaling the availability of different reward magnitudes. Here, fast-scan cyclic voltammetry was used to compare rapid dopamine release dynamics in the NAc subregions during a magnitude-based decision-making task. Rats learned that distinct cues signaled the availability of either a small or large reward (one or two sugar pellets), and then were given an opportunity to choose their preferred option. We found that peak dopamine release tracked the more preferred (higher-magnitude) option in both core and shell subregions. Critically, however, overall (i.e., global) dopamine release was significantly higher and longer lasting in the shell and tracked the preferred magnitude during the entire cue period. Further, in the shell (not core), dopamine signaling significantly declined immediately at the lever press for reward but increased during the period of reward consumption. Collectively, the results indicate that although dopamine release in both the core and shell are activated by cues signaling the opportunity to respond for rewards of different magnitudes, dopamine release in the shell plays a differential and unique role in tracking information related to the outcome value of reward.

## Significance Statement

The nucleus accumbens (NAc) has been shown to facilitate optimal decision-making; however, the precise role of its primary subregions (shell and core) in outcome-based decision-making is unclear. Here, using voltammetric recording methods in behaving rats, we show preferential real-time dopamine signaling within the NAc shell, compared with the core, during a well-learned magnitude-based decision-making task. Collectively, these findings demonstrate that dopamine in the NAc shell plays a unique role in tracking information related to reward outcome value.

## Introduction

Effective decision-making depends on an organism’s ability to predict the outcome of its choices and bias behavior toward the option of greatest value. Value-based decision-making recruits the mesolimbic system, including the nucleus accumbens (NAc) and its dopaminergic input (Day et al., 2007; Fields et al., 2007; Clark et al., 2012). Electrophysiology studies show that dopamine neurons increase activity to reward-predictive cues and track choice behaviors related to a range of decision-making tasks involving, for example, effort, delay, risk, and delay discounting (Schultz, 1997; Roesch et al., 2007). Importantly, rapid dopamine release in the NAc reflects this pattern of neural activity (Day et al., 2010; Wanat et al., 2010; Sugam et al., 2012; Saddoris et al., 2015b). Indeed, increases in transient dopamine release have been measured during cues predicting food, liquid, cocaine, and intracranial self-stimulation (Phillips et al., 2003; Roitman et al., 2004; Day et al., 2007; Beyene et al., 2010; Cacciapaglia et al., 2012; Owesson-White et al., 2008, 2016). Further, pharmacological disruptions or lesions of mesolimbic circuitry, including the NAc, result in maladaptive decision-making such that animals cannot update behavior to reflect changes in reward value (Cardinal et al., 2001; St Onge and Floresco, 2009; Ghods-Sharifi and Floresco, 2010).

The NAc contains two primary subregions, the shell and core, that differ in their afferent and efferent connections (Heimer et al., 1991; Zahm and Brog, 1992; Zahm and Heimer, 1993; Jongen-Relo et al., 1994; Corbit et al., 2001; Ikemoto, 2007) and subserve different functional properties (Carelli, 2004; Kelley, 2004; Saddoris et al., 2013, 2015a; Zorrilla and Koob, 2013; Castro et al., 2015). Importantly, studies suggest that the shell and core may encode different aspects of value-based decision-making. For example, lesion and pharmacological inactivation studies link the NAc core to subjective-based decision-making (Cardinal et al., 2001; Cardinal and Cheung, 2005; Cardinal and Howes, 2005; Pothuizen et al., 2005; Hauber and Sommer, 2009; Ghods-Sharifi and Floresco, 2010). In support, studies have shown that rapid dopamine release in the core may bias encoding toward subjective preferences in tasks involving delay, risk, effort, and delay discounting (Day et al., 2010; Sugam et al., 2012; Saddoris et al., 2015b). Conversely, dopamine activity in the shell appears to encode reward outcome, such as objectively larger reward magnitudes (Beyene et al., 2010; Stopper and Floresco, 2011; however, see Nasrallah et al., 2011). Indeed, cues predicting larger (versus smaller) reward magnitudes elicit greater dopamine cell firing (Tobler et al., 2005; Roesch et al., 2007) and higher shell dopamine release (Beyene et al., 2010).

Although these studies link the shell and core to specific aspects of decision-making, no reports have directly compared dopamine release dynamics in these subregions to cues signaling the availability of different reward magnitudes. Here, we used fast-scan cyclic voltammetry (FSCV) to examine how rapid dopamine signaling in the NAc core versus shell encodes information about cues during a magnitude-based decision-making task. Rats learned that distinct cues signaled the availability of either a small or large reward (one or two sugar pellets), and then were given an opportunity to choose their preferred option. We found that peak dopamine release tracked the more preferred (higher-magnitude) option in both core and shell subregions. Critically, however, global dopamine release dynamics were significantly higher and longer lasting in the shell and tracked the preferred reward magnitude during the entire cue period. Further, the shell, but not core, exhibited unique dopamine signaling properties relative to the lever press for reward. These findings indicate that cues signaling the opportunity to respond for rewards of different magnitudes activate dopamine release in both the NAc core and shell; however, dopamine release in the shell plays a differential and unique role in tracking information related to reward value.

## Materials and Methods

### Animals

Singly housed male Sprague-Dawley (Harlan, *n* = 12) rats were ∼90–120 d old and weighed 275–330 g at the start of experiments. Animals were maintained at no less than 85% of pre-experimental body weights by food restriction, except during the postoperative recovery period, when food was given *ad libitum* (Harlan Lab Chow). Water was available *ad libitum* throughout the duration of the experiment. Animal procedures were conducted in accordance with the National Institutes of Health Guidelines for the Care and Use of Laboratory Animals, and were approved by the University of North Carolina, Chapel Hill Institutional Animal Care and Use Committee.

### Apparatus

Behavioral testing was conducted in 43 × 43 × 53 cm Plexiglas chambers housed in sound-blocking boxes (Med Associates) described elsewhere (Saddoris et al., 2015b). Briefly, one side of each chamber was equipped with two retractable levers (Coulbourn Instruments) 17 cm apart, with a stimulus light 6 cm above each lever. Sucrose pellets (45 mg) were delivered to a food receptacle, which was located equidistant from each lever. A house light (100 mA) was mounted on the opposite side of the chamber.

### Behavioral procedures

All behavioral experiments were conducted at least 1 wk postsurgery, and rats underwent similar pretraining before beginning each behavioral task. Here, rats were trained to press two distinct levers in which each response was reinforced on a continuous schedule of reinforcement. Reinforced responses resulted in the delivery of a sucrose pellet to a centrally located food cup. Animals were trained to a criterion of 50 presses on each response lever before moving to the behavioral tasks outlined below.

Next, rats were trained on a task that involved three types of contingencies (30 trials each) intermixed within 90 total trials per session. At this stage, only a single sucrose pellet was available for each lever press throughout the session. The first two trial types were classified as forced-choice trials. For one trial type, a single cue light was illuminated for 5 s over one lever, followed by extension of both levers. Responses on the cue light illuminated lever (within 15 s) were immediately reinforced with one sucrose pellet. During the other forced-choice trial type, the cue light over the other lever was illuminated for 5 s, followed by extension of both levers. Responses within 15 s on the cue-associated lever were reinforced as above. For both forced-choice trials, responses on the unsignaled lever were counted as errors and resulted in termination of the house light for the remainder of the trial period, with no reward delivery. During the third trial type, termed free choice trials, both cue lights were illuminated for 5 s, after which both levers were extended, and responses on either lever within 15 s were reinforced with one sucrose pellet. After a press on either lever, both levers were retracted and a sucrose pellet was immediately delivered into the food receptacle. To move on to the next phase of training, rats needed to maintain at least 3 d of stable accuracy (80% correct responses).

**Fig. 1.**
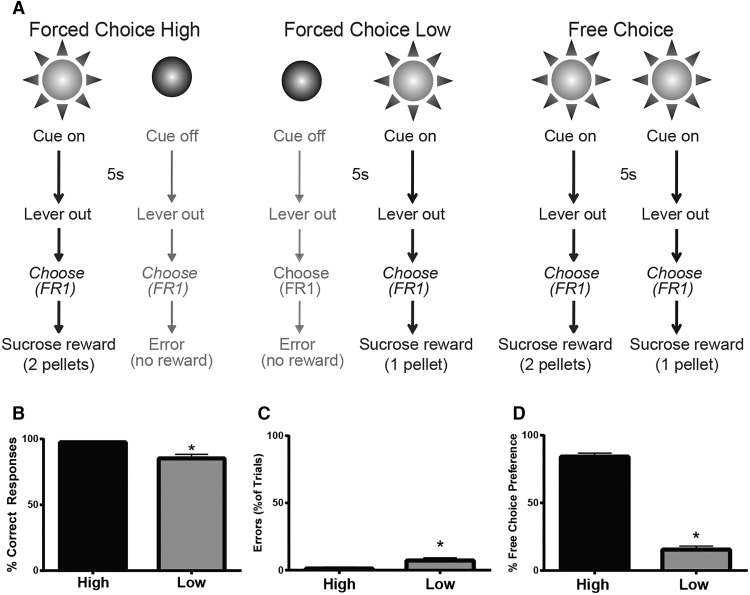
Magnitude-based decision-making task and behavior. ***A***, Schematic of behavioral task. Left, during forced choice high-magnitude trials, one cue light was illuminated for 5 s followed by extension of both levers. Presses on the correct lever resulted in two sucrose pellets. Presses on the nonsignaled lever were counted as errors, resulting in termination of the houselight for the remainder of the trial period, with no reward delivery. Middle, on forced choice low-magnitude trials, the other cue light was illuminated for 5 s followed by extension of both levers. If the correct lever was chosen, one sucrose pellet was delivered. Presses on the nonsignaled lever were counted as errors and were unrewarded. Right, during free choice trials, both cue lights illuminated for 5 s followed by extension of both levers. Responses were rewarded based on the contingency of the lever chosen. ***B***, Percentage of correct reinforced responses during high- and low-magnitude forced choice trials. ***C***, Percentage of total errors on forced choice trials. ***D***, Responses during free choice trials indicate that rats significantly preferred the high-magnitude (compared with low-magnitude) option. Data are mean ± SEM. *, *p* < 0.01.

After reaching accuracy criteria, the reward contingency on one of the levers was altered to reflect the reward magnitude decision-making task. A schematic diagram of the final task design is shown in Fig. 1*A*. Here, the task remained identical to that described above except the reward contingency on one of the levers was changed to two sucrose pellets, and responses on the other lever remained at one sucrose pellet. These assignments were counterbalanced across animals and remained constant for each rat throughout training. Animals were trained on the reward magnitude task until accuracy was stable (80% correct responses) and a clear preference (at least 60% responding on one lever during free choice trials) was observed. After acquisition of stable responding and magnitude preference, all rats were prepared for electrochemical recording in either the NAc core or shell (described below). After recovery, animals underwent additional training sessions until behavior reached presurgery baseline levels (at least three sessions).

### Surgery

Rats were deeply anesthetized with a mixture of ketamine hydrochloride (100 mg/kg) and xylazine hydrochloride (10 mg/kg), placed in a stereotaxic frame, and surgically prepared for voltammetric recording as described elsewhere (Day et al., 2010; Sugam et al., 2012; Saddoris et al., 2015b). A guide cannula (Bioanalytical Systems) was positioned dorsally to the NAc core (AP +1.3 mm, ML –1.3 mm from bregma) or shell (AP +1.3 mm, ML –0.8 mm from bregma). Another guide cannula (for the Ag/AgCl reference electrode) was placed contralateral to the NAc cannula. A bipolar stimulating electrode was placed dorsally to the ventral tegmental area (VTA; AP –5.2 mm, ML –1.0 mm and DV –7.0 mm from bregma) and ipsilateral to the NAc cannula. Correct placement of the stimulating electrode in the VTA was determined by applying a range of stimulation parameters (12–24 biphasic pulses, 20–60 Hz) and observing tail movement. The stimulating electrode was lowered in increments of 0.1 mm until slight to no tail movement was observed at 60 Hz, 24 pulses. Stainless steel screws and dental cement were then used to secure all items. For 2 d postsurgery, rats were given an anti-inflammatory medication (meloxicam, 1 mg/kg) and were allowed access to food and water *ad libitum.*

### Fast scan cyclic voltammetry

One week after surgery, rats were food-restricted and retrained on the magnitude task until they reached presurgery performance (maximum of five sessions). Changes in dopamine concentration during the task were assessed using FSCV as described elsewhere (Day et al., 2010; Sugam et al., 2012; Saddoris et al., 2015b). On the test day, a carbon-fiber microelectrode was lowered into the NAc core or shell with a locally constructed microdrive, after placing an Ag/AgCl reference electrode in the contralateral hemisphere. The carbon-fiber microelectrode was held at –0.4 V versus the Ag/AgCl reference electrode. Periodically, a cyclic voltammogram was acquired (100-ms intervals) by applying a triangular wave form that drove the potential to 1.3 V and back to –0.4 V. Changes in current at the oxidation potentiation for dopamine were compared with electrically stimulated dopamine release at the same location. Chemometric analysis was used to identify dopamine concentrations using HDCV software (UNC Chemistry Electronics) and aligned to behavioral events (Trans IV, Med Associates). In a subset of rats (*n* = 3), after recording a full session of 90 trials, the electrode was lowered another ∼300 µm until another release site was found, at which point another recording was taken for a session of 90 trials.

### Histology

After completion of the experiments, rats were deeply anesthetized with a mixture of ketamine (100 mg/kg) and xylazine (10 mg/kg). To mark the placement of the electrode tip, a tungsten electrode housed in a micromanipulator was lowered to the recording site, and a small electrolytic lesion was made. Brains were then extracted, sliced on a freezing cryostat, and placed onto slides. The location of electrode tips was assessed by visual examination of successive coronal sections in comparison to visual landmarks and the anatomic organization of the NAc core and shell, as represented in a stereotaxic atlas.

### Data analysis

All statistical analyses are reported in Table 1. Analysis of behavior during the reward magnitude task included examination of response allocation, number of errors, and free choice preference. To determine whether rats reliably acquired the task, we evaluated the number of errors and correct responses during training and test sessions. For behavioral analysis of forced choice trials during test sessions, paired *t* tests were used to compare accuracy (percentage rewarded trials) and percentage errors during high and low reward trial types. For free choice trials, a paired *t* test was used to compare reward magnitude preferences.

**Table 1.** Statistical analyses

Location	Fig.	Data structure	Type of test	Statistical value	*p*-value
a	1*B*	Normal distribution, 2-tailed	Paired *t* test	*t_14_* = 3.726	<0.01
b	1*C*	Normal distribution, 2-tailed	Paired *t* test	*t_14_* = 3.769	<0.01
c	1*D*	Normal distribution, 2-tailed	Paired *t* test	*t_14_* = 13.90	<0.001
d	3*A*	One factor (time)	One-way ANOVA	*F*_5,35_ = 3.214	<0.05
			Fisher’s LSD	High Low Choice	<0.05 >0.05 <0.05
		Normal distribution, 2-tailed	Paired *t* test	*t*_5_ = 1.779	>0.05
		Normal distribution, 2-tailed	Paired *t* test	*t*_7_ = 0.3043	>0.05
e	3*B*		1-Way ANOVA	*F*_5,30_ = 0.6477	>0.05
		Normal distribution, 2-tailed	Paired *t* test	*t*_4_ = 1.006	>0.05
		Normal distribution, 2-tailed	Paired *t* test	*t*_5_ = 0.6448	>0.05
f	4*A*	Two factors (trial type, region)	2-way ANOVA	Trial type: *F*_2,26_ = 14.40 Region: *F*_1,13_ = 5.124 Interaction: *F*_2,26_ = 2.371	<0.0001 <0.05 0.1133
			Bonferroni’s	High vs. low High vs. choice Choice vs. low	<0.05 >0.05 <0.05
g	4*B*	Two factors (trial type, region)	2-way ANOVA	Trial type: *F*_2,26_ = 7.584 Region: *F*_1,14_ = 2.738 Interaction: *F*_2,14_ = 0.857	<0.01 0.1219 0.4362
			Bonferroni’s	High vs. low High vs. choice Choice vs. low	<0.05 >0.05 <0.05
h	5*B*	Two factors (trial type, region)	2-way ANOVA	Trial type: *F*_2,24_ = 0.42 Region: *F*_1,12_ = 5.744 Interaction: *F*_2,24_ = 0.026	0.6617 <0.05 0.9748

Analysis of FSCV recordings was similar to that of previous reports (Day et al., 2010; Sugam et al., 2012; Saddoris et al., 2015b). Briefly, each subject received electrical stimulation of VTA afferents (frequency, 12–60 Hz; pulses, 1–20) to generate a training set of dopamine release at the recording location in the NAc. To analyze recorded FSCV data, each subject’s training set collected from the site of recording was used to chemometrically convert recorded current during the session into dopamine concentrations (Rodeberg et al., 2015). Concentrations were then aligned to behavioral events to assess dopamine-release dynamics relative to task stimuli.

Because free choice trials allowed rats to choose a large-versus-small sucrose reward, there were unequal numbers of responses to high- versus low-magnitude options. Thus, because of differences in variance, it would be inappropriate to compare dopamine release for the average of 26 high-magnitude choices compared with 4 low-magnitude choices. Therefore, we combined all dopamine concentrations for the 30 free choice trials, regardless of what option the rat eventually chose. To confirm this approach, we randomly selected an equal number of free choice trials in which animals responded to the low versus high choice (i.e., on average, 4 high choice trials to 4 low choice trials) and compared peak dopamine release during these trials using paired *t* tests. Likewise, we used a similar approach to examine cue-evoked dopamine release during forced choice correct versus incorrect (i.e., error) trials. Here, we included equal numbers of correct and incorrect trials (i.e., on average, 4 correct versus 4 incorrect trials) and compared peak dopamine release using paired *t* tests.

To assess the differential effects of the three cue types on dopamine release in both core and shell, peak dopamine concentrations within 2 s following cue presentation were analyzed using a two-way ANOVA with Bonferroni’s multiple comparison test. To compare amounts of dopamine release in the shell versus core, we examined the cumulative dopamine release in each region by summing the concentration of dopamine in each bin during 0.5–4 s after cue onset for the forced and free choice trials to provide an estimate area under the curve and completed a two-way ANOVA with Bonferroni’s multiple comparison test on these data. To examine dopamine release dynamics relative to the lever press, we examined the depth of the dopamine “trough” after the press. This was analyzed by taking the minimum dopamine concentration within 2 s after lever press in the core and shell and performing a two-way ANOVA. To analyze dopamine release after lever press, a one-way ANOVA with Fisher’s LSD multiple comparison test was performed comparing dopamine concentration at the peak trough time point compared with 2 s afterward at the peak of the increase.

All analyses were considered significant at α = 0.05. Statistical and graphical analysis were performed using GraphPad Prism 6.0 for Windows (GraphPad Software) and Neuroexplorer for Windows version 4.034 (Plexon Inc.). Statistical outliers were calculated using extreme studentized deviate test and excluded from further analysis.

## Results

### Behavior

Rats rapidly learned the magnitude decision-making task and discriminated between the cue types. Specifically, rats completed significantly more correct responses during high forced choice trials (Fig. 1*B*, *t*_14_ = 3.726, *p* < 0.01^a^) and made significantly more errors (Fig. 1*C*, *t*_14_ = 3.769, *p* < 0.01^b^) during low forced choice trials. On free choice trials, all rats exhibited a significant preference (>60% choice) for the high-magnitude option over the low-magnitude option (Fig. 1*D*, *t*_14_ = 13.90, *p* < 0.001^c^).

### Differential dopamine release patterns in the NAc shell versus core to reward predictive cues

**Fig. 2.**
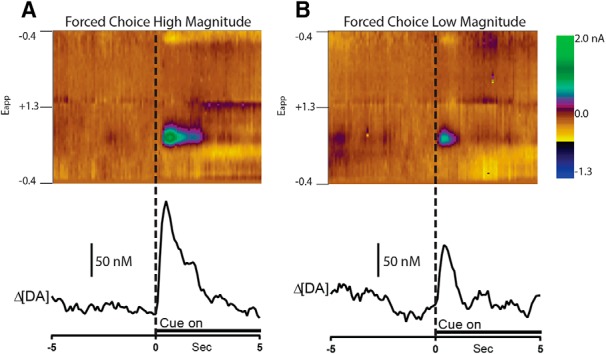
Example of differential dopamine release dynamics to the cue signaling high and low forced choice trials. Two-dimensional color representations of cyclic voltammetric data collected for 10 s around forced high-magnitude (***A***) and forced low-magnitude (***B***) trials in the NAc shell for a single representative animal. The ordinate is the applied voltage (Eapp) and the abscissa is time (s). Differential dopamine concentration [DA] determined via principal component analysis is plotted below the color plots.

Reward-predictive cues evoked rapid dopamine release in the NAc shell and core. However, forced choice high-magnitude trials induced higher concentrations of dopamine release than forced choice low-magnitude trials. This finding is illustrated for a representative animal in Fig. 2. Here, color representation of a set of background-subtracted cyclic voltammograms and the corresponding dopamine concentration trace are averaged across all forced high and forced low trials of a single session. During the forced high-magnitude trial (left), the onset of the cue (indicated by dashed line at time 0) resulted in an increase in rapid dopamine release that reached a maximum of ∼170 nm. Although a similar increase in NAc dopamine release was observed during forced low trials, it was of lower concentration (∼100 nm).

**Fig. 3.**
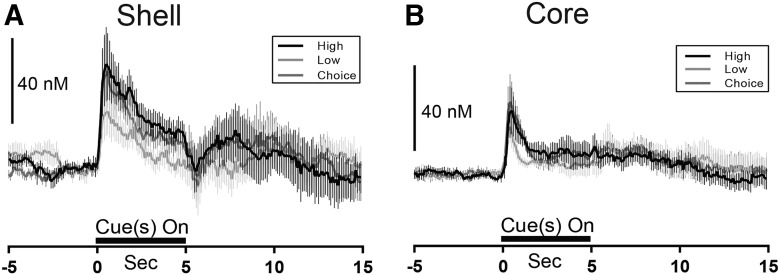
Dopamine release dynamics in the NAc shell and core during the magnitude based decision-making task. ***A***, Average dopamine release in the shell across all rats and trial types. ***B***, Average dopamine release in the core across all rats and trial types. Dashed line at time 0 indicates cue period in ***A*** and ***B***.

**Fig. 4.**
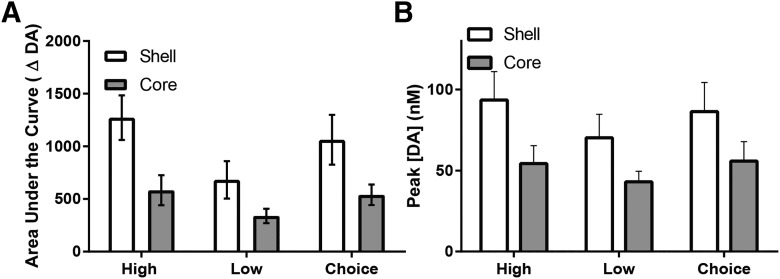
Cue-evoked dopamine release dynamics in the NAc shell and core. ***A***, Area under the curve analysis across both the shell and core during forced high, forced low, and free choice trials. ***B***, Peak dopamine concentration [DA] across all rats in the shell and core during forced high, forced low, and free choice trials. All data are mean ± SEM. *, *p* < 0.05.

Fig. 3*A* and *B* shows average dopamine release dynamics in the shell and core across all animals during the task. Forced choice trials in which rats made incorrect responses (i.e., errors) were excluded from analysis, as peak dopamine release was not significantly different from correct (reinforced) responses in the shell (*t*_5_ = 1.779, *p* > 0.05^d^) or core (*t*_4_ = 1.006, *p* > 0.05^e^). Free choice trials were combined regardless of rats’ eventual choice, as random sampling analysis indicated no difference in dopamine release during trials in which rats chose the high versus low option in either the shell (*t*_7_ = 0.3043, *p* > 0.05^d^) or core (*t*_5_ = 0.6448, *p* > 0.05^e^). It is immediately apparent that although dopamine was released to cues in both NAc subregions, it was generally higher in concentration in the shell (Fig. 3*A*, left) than core (Fig. 3*A*, right). To quantify this finding, we examined the cumulative dopamine release in each region by summating the concentration of dopamine in each bin during 0.5–4 s after cue onset for the forced and free choice trials to provide an estimate area under the curve (Fig. 4*A*). Two-way ANOVA revealed a main effect of trial type (*F*_2,26_ = 14.40, *p* < 0.05^f^) and a main effect of region (*F*_1,13_ = 5.124, *p* < 0.05^f^), but importantly, no significant interaction between region and trial type (*F*_2,26_ = 2.371, *p* > 0.05^f^). These findings confirm that although all cue types significantly increased dopamine release in both regions, release was significantly higher in the NAc shell, compared with core. Further, dopamine signaling during high forced choice and free choice cues was significantly higher than during low forced cues across both subregions (*p* < 0.05^f^).

Reward-predictive cues also evoked significant increases in peak dopamine release in both subregions, as illustrated in Fig. 4*B*. Two-way ANOVA revealed a significant main effect of trial type (*F*_2,26_ = 7.584, *p* < 0.01^g^) but no significant main effect of region (*F*_1,14_ = 2.738, *p* > 0.05^g^) or interaction (*F*_2,14_ = 0.857, *p* > 0.05^g^). *Post hoc* tests on the main effect of trial type revealed higher peak dopamine release for high and choice trials, compared with low magnitude trials, independent of region (*p* < 0.05^g^). High and choice trial peak dopamine were not different from each other (*p* > 0.05^g^).

### Differential dopamine release dynamics in the NAc shell versus core at lever press

**Fig. 5.**
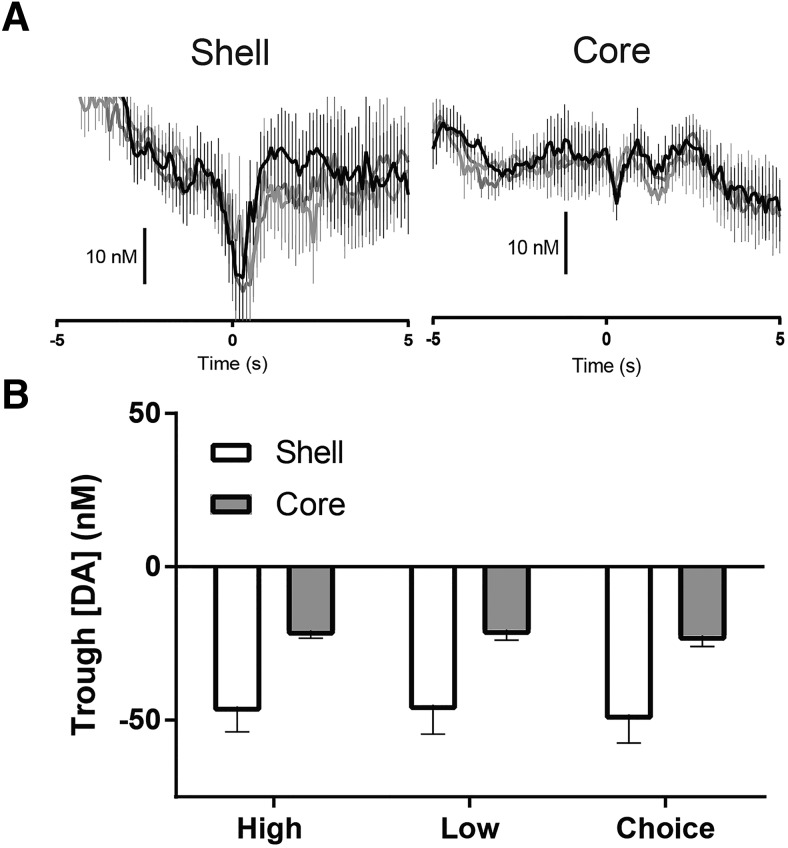
Dopamine release dynamics relative to the lever press response during the task. ***A***, Dopamine release dynamics in the NAc shell and core aligned to lever press response (onset at time 0) across forced high, forced low, and free choice trials. ***B***, Trough dopamine concentration [DA] across all rats in the shell and core during forced high (black lines), forced low (light gray lines), and free choice (dark gray lines) trials. All data are mean ± SEM. *, *p* < 0.05.

Five seconds after cue presentation, both levers were extended into the chamber and rats could press a single lever indicating either one reward outcome or no reward (forced choice trials) or choice of high versus low reward options (free choice trials). In the shell, a pronounced dip in dopamine release occurred after lever press (Fig. 5*A*, left). In comparison, this decrease in dopamine release was less pronounced in the core (Fig. 5*A*, right). To further examine this finding, we quantified the lowest point (i.e., trough) in dopamine release after the lever press across both subregions (Fig. 5*B*). Two-way ANOVA on mean trough dopamine revealed a main effect of region (*F*_1,12_ = 5.744, *p* < 0.05^h^) but no main effect of trial type (*F*_2,24_ = 0.42, *p* > 0.05^h^) or interaction (*F*_2,24_ = 0.026, *p* > 0.05^h^). These findings indicate that the dip in dopamine evident after the lever press was most pronounced in the shell, regardless of trial type. Interestingly, in the shell, a subsequent increase in peak dopamine release was observed within 2 s after the lever press (*F*_5,35_ = 3.214, *p* < 0.05^d^), but only in forced high and free choice trials (*p* < 0.05^d^). This increase was not observed in the core (*F*_5,30_ = 0.6477, *p* > 0.05^e^).

### Histology

**Fig. 6.**
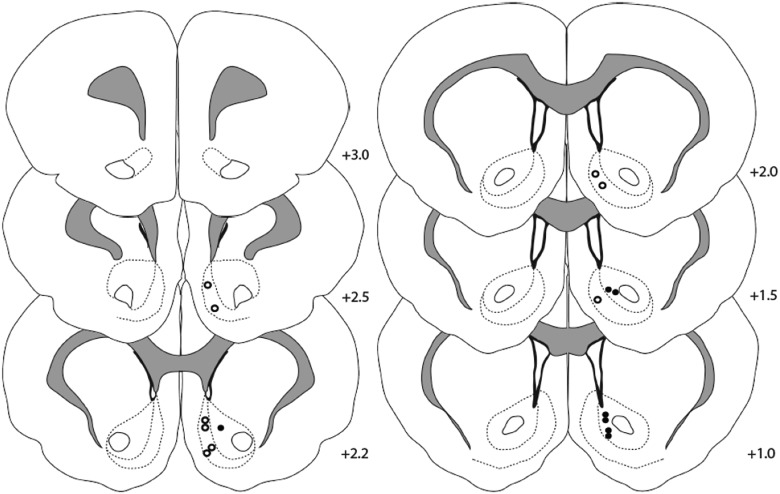
Histologic confirmation of carbon-fiber microelectrodes tips in the NAc shell (*n* = 8, white circles) or core (*n* = 7, black circles).

Placement of carbon fiber electrode tips used in FSCV experiments were confirmed to be in the NAc core or shell (Fig. 6).

## Discussion

In the present study, electrochemical methods were used in rats to compare dopamine signaling dynamics in the NAc shell and core during a magnitude-based decision-making task in which discrete cues signaled the availability of different reward sizes (one vs. two sugar pellets). Although similar cue-associated peak dopamine release events were observed across the two NAc subregions, higher dopamine concentrations that extended throughout cue periods, and tracked predicted magnitude information was exclusively measured in the shell. Further, in the shell (not core), there was a significant decline in dopamine signaling immediately after the lever press for reward followed by a significant increase in dopamine release at the time of reward consumption. Collectively, the present findings provide novel insight into rapid dopamine signaling dynamics across NAc subregions and reveal a unique role of the shell in tracking information about expected outcome value.

Elements of value-based decision-making may be separable into two interrelated but distinct components (Saddoris et al., 2015b), including outcome-based features of the association (i.e., reward magnitude), as well as subjective costs that may be more variable across individuals (e.g., willingness to engage in risky behaviors). The NAc shell and core appear to differentially contribute to outcome versus subjective-based decision-making. For example, it has previously been reported that peak dopamine in the NAc core, but not shell, plays a more discrete role in encoding how subjective costs such as delay, effort, and risk can modulate the value of anticipated rewards (Day et al., 2010; Sugam et al., 2012; Saddoris et al., 2015b). For example, Sugam et al. (2012) found that the NAc core tracks reward prediction error during a risky decision-making task when rats expected a positive reward outcome but did not receive it. Conversely, dopamine release in the NAc shell scales with cues signaling differences in reward magnitude (Beyene et al., 2010) consistent with its role in magnitude processing (e.g., Stopper and Floresco, 2011; Bassareo et al., 2015), but was much less sensitive to subjective costs such as effort, delay, or risk tolerance (Day et al., 2010; Sugam et al., 2012). The present study is consistent with those reports by revealing differential dopamine release dynamics across NAc subregions with unique signaling features related to outcome value in the shell. Further, the current findings support the assertion that whereas the core tracks reward prediction errors, the shell evaluates the incentive salience of an outcome (as described in detail in Saddoris et al. [2015a]).

Some previous studies have reported that cue-associated rapid dopamine release in the NAc core encodes differences in reward magnitude (Gan et al., 2010; Saddoris et al., 2015b). However, in those studies, the reward magnitude feature was embedded in more complex tasks that recruited both subjective costs and outcome-based components (e.g., delay discounting). Interestingly, Nasrallah et al. (2011) reported magnitude-related differences in dopamine signaling in the core, although that report involved “free” unsignaled rewards of different sizes. However, in a recent report, it was shown that core dopamine signals did not discriminate between unsignaled deliveries of either a small (one pellet) or large (two pellets) reward (Saddoris et al., 2017). Regardless, by incorporating the magnitude-based decision-making task used here, we were able to tease apart aspects of cue-associated dopamine signaling to rewards of different sizes and directly compare release events across the shell and core not examined previously.

Using this comparative approach, we were able to reveal that although peak dopamine release to cues was similar across the shell and core, overall dopamine release was significantly larger and longer lasting in the shell (i.e., was evident up to the lever press response), compared with the core. Further, a brief yet significant dip in dopamine release where concentrations extended down to baseline (i.e., the differential dopamine concentration before the cue) was evident immediately after the lever press in the shell during all trial types (forced and free choice trials). This significant decline in dopamine release was not observed in the core. Although it is not known exactly what this postresponse drop in dopamine release reflects, it may be indicative of a behavioral shift from food seeking to consummatory behavior. Indeed, electrophysiological studies have shown that neurons in the NAc shell, but not core, were inhibited on lever press for a reward (Ambroggi et al., 2011), and that a pause in NAc cell firing is required to initiate and maintain feeding behaviors (Krause et al., 2010). Further, we also observed that after this dip in dopamine (i.e., during the reward delivery and consummation period), a significant increase in dopamine was observed in the shell (not core). This increase was significant during high forced and free choice trials, in which animals usually received a large-magnitude reward. As such, the dopamine release (and pause) dynamics observed in the present study could be attributed to components of the shell’s unique “feeding” circuit (Stratford and Wirtshafter, 2012).

The present findings are also consistent with previous studies implicating the shell in encoding reward outcome (Beyene et al., 2010; Stopper and Floresco, 2011; Bassareo et al., 2015; Saddoris et al., 2017) and complement previous literature linking this subregion to reward hedonics and valence (Pecina and Berridge, 2000, 2005; Kelley, 2004; Zorrilla and Koob, 2013; Castro et al., 2015; Saddoris et al., 2015a). For example, glutamate antagonists microinfused into the shell enhanced appetitive behavior in rats (Maldonado-Irizarry et al., 1995; Kelley and Swanson, 1997), whereas NAc shell inactivation slightly reduced rats’ preference for large versus small rewards (Stopper and Floresco, 2011) and core and shell neurons differentially encoded cues signaling availability of rewards (Ambroggi et al., 2011). Further, novel, uncued delivery of appetitive food-related stimuli evoked increases in NAc shell dopamine (Bassareo and Di Chiara, 1997, 1999; Roitman et al., 2008; Wheeler et al., 2011). These findings are consistent with the view that the shell is biased toward processing both consummatory and associative information about reward value, which likely updates relative to the animal’s motivational state (Floresco et al., 2008; Corbit and Balleine, 2011; Saddoris et al., 2015a).

Our findings are also consistent with previous work by Cacciapaglia et al. (2012) that demonstrated dopamine release to sucrose-associated cues that was larger and longer lasting in the NAc shell compared with the core. In that study, dopamine release was measured in a task involving pressing a single lever after cue onset, for a single sucrose pellet. Critically, the present study extends that report by examining distinct aspects of dopamine signaling during a task in which cues signaled different reward magnitudes. In both studies, dopamine release peaked during cues that signaled reward in both subregions and was prolonged in the shell throughout the cue. Here however, by incorporating a task that enabled the ability to choose high (two pellets) and low (one pellet) options, we also show that rapid dopamine release tracks the predicted outcome during this prolonged signaling. This variance in dopamine signaling across subregions may be attributed to functional and anatomic differences between the core and shell (Ikemoto, 2007). For example, there is lower dopamine uptake in the shell (compared with core; Jones et al., 1996), which may explain, in part, the prolonged dopamine concentration measured in the shell in the present study. This prolonged synaptic presence of dopamine in the shell could facilitate a role of this subregion in tracking preferred reward outcome and inform downstream structures about reward availability. This unique signaling could then influence the selection of the best value choice (i.e., choose the largest available food source when possible).

In conclusion, the present study shows that peak cue-evoked dopamine release in both core and shell subregions scaled to information related to rewards of different magnitudes. However, throughout the entire 5-s cue duration, shell dopamine continued to track magnitude information and remained increased throughout the entire cue period, reflecting the expected value of the predicted reward. Further, a pause in dopamine signaling was observed in the shell (not core) immediately at the lever press for reward, followed by a brief increase in dopamine release during reward delivery and consumption. This unique signal pattern likely relates to the shell’s unique role in consummatory behaviors. Collectively, the results support the view that the core encodes individual, preferential value and is better linked to tracking simple outcome value. Future studies (e.g., using optogenetics) are needed to determine whether the unique dopamine signaling properties in the NAc shell observed in this study are causally linked to behaviors involving simple decisions to respond for rewards of different magnitudes.

## Synthesis

Reviewing Editor: Karen Szumlinski, University of California at Santa Barbara Decisions are customarily a result of the Reviewing Editor and the peer reviewers coming together and discussing their recommendations until a consensus is reached. When revisions are invited, a fact-based synthesis statement explaining their decision and outlining what is needed to prepare a revision will be listed below. The following reviewer(s) agreed to reveal their identity: James Jentsch, Stan Floresco.

In consultation with 2 reviewers with expertise in dopamine physiology as it relates to goal-directed behavior, we have reached the decision of “Revise and Re-review” for your research article submitted to eNeuro. Both of the reviewers and I agree that the findings summarized in your report are very straight-forward and that the clever, novel, model presented advances the field. However, both reviewers and I agree that while the analyses conducted were appropriate and well-discussed, additional analyses of the existing data could provide greater insight into the relation between the dopamine signal and behavior.

More specifically, Reviewer 1 suggested a sub-analysis of the data on a trial by trial basis, given that on certain trials that were signaled as “low” reward, the rats selected the high reward lever and vice versa. Reviewer 1 felt that although the frequency of these two types of events were very different, it would be important to evaluate whether differences in cue-evoked dopamine output reliably predicted a correct or incorrect choice. Such analyses would then enable a discrimination of the components of dopamine release that reflect the associative information of the cue from the response selection process. Reviewer 1 acknowledges that the number of incorrect trials may be too uncommon to conduct this sort of sub-analysis and if that it the case, requests that some discussion of this issue at least be discussed. I concur with this suggested sub-analysis.

Reviewer 2 suggested other follow-up analyses based on the data presented in Figure 3. Given that dopamine levels in the shell (but not the core) exhibited a secondary increase during the 5-10 sec epoch when the reward was delivered, this reviewer requests that an analysis of the dopamine trace during reward delivery also be included for both subregions. Furthermore, this reviewer requests that the outcome(s) of this analyses be discussed with respect to the findings of Sugam et al (2012), in which animals performed a more complex risk/reward decision making task and observed a change within the core. Any such differences between studies are important in understanding the conditions where receipt of reward can (or cannot) cause changes in dopamine release. Reviewer 2 also argues that if increases in the shell are still significant from baseline, then this is another important difference between regions. Further, even if no differences are apparent, it is important to point out that when animals know that actions will always yield some rewards, the impact of these rewards on dopamine transmission is minimal (at least in the core). I concur with this suggested re-analysis.

Reviewer 2 also argues that, in the discussion, (pg 13, 1st paragraph), the statement: “ the present findings ...reveal a unique role of the shell in tracking information about outcome value” is not quite correct given that dopamine was analyzed with respect to the expected value of the reward and not the “outcome” value. This issue would be resolved with the inclusion of the reviewer's suggested supplemental analysis.

Reviewer 2 also suggested that one way in which the authors might consider analyzing the dopamine data on the free-choice trials when rats selected the large vs small reward could be to randomly select data from a number of trials when animals chose the large reward so that an equal number of trials for both large and small reward selections are used. This reviewer argued that if there is a difference, that would be of interest, but it would be even more interesting to show no difference in how these signals differ even when action selection is different. If this analysis is not possible, this review would like to see a display of these data for the core and shell, perhaps as another set of panels in Figure 3? I agree with this suggestion and the alternative should it prove impossible to attempt this analysis.

There were also a few more minor issues noted by Reviewer #2:

1)Pg 15: the authors stated “NAc shell inactivation disrupts the ability of rats to judge reward magnitude options (Stopper and Floresco, 2011)”. This is not the best way to phrase these results- the cited study showed that NAc shell inactivation caused a slight reduction in preference to choose larger vs smaller rewards. It is unclear whether this is specifically related to value judgements (and see Floresco, 2015, Annual Reviews Psycholgoy for additional interpretation of what may be driving this effect). This should be rephrased.

2) Pg 12: some awkward wording: “In the shell, a pronounced dip in dopamine release occurred following lever press (Figure 5A, left), which was greatly attenuated in the core (Figure 5A, right).” Probably better to split this into two sentences like “In the shell, a pronounced dip in dopamine release occurred following lever press (Figure 5A, left). In comparison, dopamine release in the core showed a considerably smaller dip in release during the lever press.” More specifically, this reviewer recommends were the case, to be the basis of this analysis, but it would be worthwhile to see some discussion of this issue in the manuscript.
